# Joint Analysis of Genome-Wide Association Data Reveals No Genetic Correlations Between Low Back Pain and Neurodegenerative Diseases

**DOI:** 10.3389/fgene.2021.744299

**Published:** 2021-09-22

**Authors:** Pengfei Wu, Bing Du, Bing Wang, Rui Yin, Xin Lv, Yuliang Dai, Wan Zhang, Kun Xia

**Affiliations:** ^1^Center for Medical Genetics & Hunan Key Laboratory of Medical Genetics, School of Life Sciences, Central South University, Changsha, China; ^2^Department of Neurology, Beth Israel Deaconess Medical Center and Harvard Medical School, Boston, MA, United States; ^3^Department of Spine Surgery, The Second Xiangya Hospital, Central South University, Changsha, China; ^4^Center for Digital Spine Surgery, Central South University, Changsha, China; ^5^Department of Biomedical Informatics and Harvard Medical School, Boston, MA, United States; ^6^Department of Biology, College of Arts & Sciences, Boston University, Boston, MA, United States; ^7^CAS Center for Excellence in Brain Science and Intelligence Technology (CEBSIT), Shanghai, China; ^8^Hengyang Medical School, University of South China, Hengyang, China

**Keywords:** low back pain, Alzheimer’s disease, Parkinson’s disease, amyotrophic lateral sclerosis, Mendelian randomization, linkage disequilibrium score regression

## Abstract

**Background:** We aimed to explore the genetic correlation and bidirectional causal relationships between low back pain (LBP) and three neurodegenerative diseases, Alzheimer’s disease (AD), Parkinson’s disease (PD), and amyotrophic lateral sclerosis (ALS).

**Methods:** Summary-level statistics were obtained from genome-wide association studies of LBP (*n* = 177,860), AD (*n* = 63,926), PD (*n* = 482,730), and ALS (*n* = 80,610). We implemented linkage disequilibrium score regression to calculate heritability estimates and genetic correlations. To investigate possible causal associations between LBP and three neurodegenerative diseases, we also conducted a bidirectional two-sample Mendelian randomization (MR) study. Inverse variance-weighted MR was employed as the primary method to generate overall estimates, whereas complementary approaches and sensitivity analyses were conducted to confirm the consistency and robustness of the findings.

**Results:** There was no evidence of genetic correlations between LBP and AD (*Rg* = −0.033, *p* = 0.766). MR analyses did not support the causal effect of LBP on AD (*OR* = 1.031; 95% CI, 0.924–1.150; *p* = 0.590) or the effect of AD on LBP (*OR* = 0.963; 95% CI, 0.923–1.006; *p* = 0.090). Likewise, this study failed to identify genetic correlations between LBP and two other neurodegenerative diseases. MR results of the associations of LBP with PD and ALS, and the reverse associations, did not reach Bonferroni-corrected significance.

**Conclusion:** The study did not support genetic correlations or causations between LBP and three common neurodegenerative diseases, AD, PD, and ALS in the European population.

## Introduction

Neurodegenerative diseases have imposed a heavy burden on the global healthcare in line with the accelerated trend of population aging. Alzheimer’s disease (AD) is the most common neurodegenerative disorder and the leading cause of dementia characterized by severe decline in cognitive function (Weller and Budson, 2018). Parkinson’s disease (PD) is the second most common neurodegenerative disease and the primary movement disorder attributed to neurodegeneration (Balestrino and Schapira, 2020). Amyotrophic lateral sclerosis (ALS), also known as Lou Gehrig’s disease, is the most common type of motor neuron disease (Hardiman et al., 2017; Liu et al., 2018). With their etiology and mechanism largely unknown, there are no effective treatments to slow down the progression of neurodegenerative diseases so far (Dorst et al., 2018; Piton et al., 2018; de Bie et al., 2020). Patients get worse gradually and lose basic activities of daily living in the last stage. With enhancement in international collaboration and advancement in genomic sciences, especially large-scale genome-wide association studies (GWAS), genetic underpinnings of neurodegenerative diseases are being elucidated (Nicolas et al., 2018; Kunkle et al., 2019; Nalls et al., 2019; Roberts et al., 2020). Low back pain (LBP) is a common health condition with escalating healthcare utilization. In the last three decades, LBP has been the leading level-3 cause of years lived with disability (YLDs) globally, and particularly in high-income countries (Vos et al., 2012; Hoy et al., 2014). According to the most recent Global Burden of Disease Study (GBD, 2020), LBP was responsible for 780 YLDs per 100,000 population, and among 692 million non-communicable disease YLDs the proportion contributed by LBP was approximately 9.2%. LBP affects all age groups with a lifetime prevalence of about 40% (Manchikanti et al., 2014), which increases with aging and is slightly higher in women (Shmagel et al., 2016). Apart from behavioral and social-economic factors, the genetic basis of LBP has been well recognized in previous studies (Livshits et al., 2011; Junqueira et al., 2014; Suri et al., 2021).

Possible relationships between LBP and neurodegenerative diseases have been previously postulated (Broetz et al., 2007; Aggarwal et al., 2010; Miller et al., 2013; Udeh-Momoh et al., 2019; Silveira Barezani et al., 2020). In a prospective cohort of 690 participants at the preclinical stage of AD (Udeh-Momoh et al., 2019), back pain was among the most frequently occurring (3.0%) safety events, whereas in a recent cross-sectional study of 115 patients with sporadic PD (Silveira Barezani et al., 2020), 58.3% of participants reported to have LBP. A higher prevalence of back pain in PD patients (75/101, 74.3%) when compared with age-matched control patients (35/132, 26.5%) was reported in another prior study (Broetz et al., 2007). With regard to ALS, back pain was also among top safety concerns (8/32, 25%) in prior clinical trials (Aggarwal et al., 2010; Miller et al., 2013). Notably, these studies had limited sample size due to ethical and economic restrictions, and unmeasured confounding and reverse causation would incur biases to the findings as well. Meanwhile, established at parental gamete formation and insusceptible to later-life environmental confounders, genetic variants precede disease onset and hence are ideal epidemiological instruments. The last two decades have witnessed great strides in GWASs (Visscher et al., 2017), particularly increased samples and augmented power, and numerous single-nucleotide polymorphisms (SNPs) have been identified for common disorders, including self-reported back pain (Freidin et al., 2019) and chronic back pain (Suri et al., 2018). From the perspective of human genomics and genetic epidemiology, cutting-edge statistical tools such as linkage disequilibrium score regression (LDSC) (Bulik-Sullivan et al., 2015; Zheng et al., 2017) and Mendelian randomization (MR) (Hemani et al., 2018; Walker et al., 2019), have made it possible to use GWAS summary-level data to explore genetic correlation (Wang et al., 2020; Zhuang et al., 2021) and make causal inference (He et al., 2020; Zhang et al., 2020) within a wide spectrum of complex traits.

In this study, we utilized LDSC to investigate genetic correlations and further conducted two-sample bidirectional MR to explore relationships between LBP and three neurodegenerative diseases.

## Materials and Methods

### Data Sources

This study was based on publicly available GWAS datasets, with informed consent from participants and approval by ethics committees completed in original studies (Nicolas et al., 2018; Kunkle et al., 2019; Nalls et al., 2019; FinnGen, 2021).

Summary association statistics for LBP was retrieved from the FinnGen study (FinnGen, 2021). LBP was defined as back pain localized between the costal margin and the inferior gluteal folds. From the Finnish registries of hospital discharge and cause of death, cases of LBP were ascertained using electronic health records with specific International Classification of Diseases (ICD) code (ICD-10, M54.5; ICD-9, 724.2; ICD-8, 728.7). Patients with symptoms of back pain caused by other specific diseases, such as fracture of lumbar vertebra (ICD-10, S32.0) and ankylosing spondylitis (ICD-10, M45), were excluded. Totally, there were 13,178 cases of LBP and 164,682 controls of the European ancestry (Supplementary Table 1). GWAS was performed in SAIGE, version 0.36.3.2 (Zhou et al., 2018), with sex, age, genotyping batches, and first 10 principal components incorporated as covariates. Variant positions which were initially presented in base pairs on build GRCh38 underwent coordinate conversion to GRCh37 using the command line tool *liftOver* and reference chain files from the UCSC Genome Browser Database (Lee et al., 2020). Effect size was reported in the unit of log-transformed odds ratio (OR) per additional copy of the alternative allele (Supplementary Table 2).

Summary-level GWAS data of three neurodegenerative diseases were from large-scale meta-analyses of AD (Kunkle et al., 2019), PD (Nalls et al., 2019), and ALS (Nicolas et al., 2018) in the European population. There were 21,982 clinically diagnosed cases and 41,944 controls in the GWAS of AD (Kunkle et al., 2019), 33,674 cases and 449,056 controls in the GWAS of PD (Kunkle et al., 2019), and 20,806 cases and 59,804 controls in the GWAS of ALS (Kunkle et al., 2019). More details of demographic information and case ascertainment were described in Supplementary Materials of original studies. GWAS meta-analyses were implemented using PLINK v1.90 (Purcell et al., 2007). Coordinates of SNPs according to the GRCh37 build were adopted; thus, no conversion was required. Likewise, the effect size represented change in log-OR of AD, PD, or ALS in the additive logistic regression (Supplementary Table 3).

### Linkage Disequilibrium Score Regression

We used the common line tool *ldsc* v1.0.1 (Bulik-Sullivan et al., 2015) to compute heritability estimates and genetic correlations from summary-level statistics. Pre-calculated reference LD scores according to the 1000 Genomes EUR panel were adopted.^1^ First, we filtered our data to keep HapMap3 SNPs (International HapMap 3 Consortium, Altshuler et al., 2010), using the recommended SNP list in the LD hub (Zheng et al., 2017). These variants had minor allele frequencies above 1% and were well-imputed in most European-ancestry GWASs, which benefited minimizing biases in the ensuing analyses. Variants at the MHC locus were not considered due to their great potential of pleiotropy and the complexity of local LD structure, which would affect the robustness of LDSC results. Those SNPs with large effect sizes (χ*^2^* > 80) were filtered, since outliers could disproportionately influence the regression. Totally, 1,160,464 SNPs for LBP, 1,204,767 for AD, 1,120,769 for PD and 1,170,115 for ALS were retained. Heritability (*H*^2^) on the observed scale, genomic inflation factor (*λ_*GC*_*), mean chi-square (χ*^2^*), and intercept statistics were derived from the SNP heritability analysis (command-line, –h2) for LBP and three neurodegenerative diseases. We divided the heritability estimate by its related standard error (SE) to calculate heritability *z*-scores. Suggested criteria (Zheng et al., 2017) to get reliable estimates of the genetic correlation were all met for LBP and three neurodegenerative diseases. The genetic correlation estimate (*Rg*) and its associated SE were computed with the −rg command flag. In the genetic correlation analysis, the *p*-value below the Bonferroni-corrected threshold (*p* < 0.05/3 = 0.017) was considered to be significant.

### Mendelian Randomization

We performed bidirectional MR using the TwoSampleMR (version 0.5.6) package (Hemani et al., 2018) in R 3.6.3 (R Foundation for Statistical Computing, Vienna, Austria). First, instrumental SNPs robustly associated with traits of interest were selected. Using the default clumping threshold (*r*^2^ < 0.001 within a 10,000 kb distance) in the MR-Base platform (Walker et al., 2019), we obtained 20, 23, and 6 SNPs associated with AD, PD, and ALS, respectively, reaching the significance threshold (*p* < 5 × 10^–8^). Regarding LBP, however, there were no genome-wide significant loci identified outside the MHC locus. Therefore, we relaxed the threshold (*p* < 5 × 10^–6^), as previous studies did (Schooling and Ng, 2019; Kwok et al., 2020; Ng and Schooling, 2020; Kwok and Schooling, 2021), to select 17 instrumental variants of LBP. For instrumental SNPs which were not present in the outcome datasets, we also searched for available proxies (*r*^2^ > 0.8, 1000 Genomes EUR). We aligned effect alleles within each exposure–outcome pair, and the harmonized and merged datasets were utilized for subsequent analyses. As the primary MR analysis, we employed the inverse variance weighted (IVW) model to compute the overall estimate (Burgess et al., 2013). The weighted median approach would provide robust estimates on the assumption that more than 50% of weights came from valid instruments (Bowden et al., 2016). MR-Egger regression was capable of examining unbalanced horizontal pleiotropy *via* the intercept and provided causal estimate with adjustment for pleiotropy *via* the regression slope (Burgess and Thompson, 2017). The weighted mode-based method would obtain a robust overall causal estimate when the majority of similar individual estimates were from valid instrumental SNPs (Hartwig et al., 2017). Nevertheless, the weighted median, MR-Egger, and weighted mode estimates had compromised power (Slob and Burgess, 2020), as indicated by wide confidence intervals (CIs), and hence were performed as complimentary methods. As for MR results, ORs represented the relative odds of the occurrence of the outcome concerned (i.e., AD) given exposure to the trait of interest (i.e., LBP). The power calculation was performed using a web application, mRnd (Brion et al., 2013). We estimated the proportion of variance explained by instrumental SNPs for the exposure using the formula 2 × EAF × (1-EAF) × Beta^2^, where EAF is the effect allele frequency and Beta denotes the effect size. Then, assuming a power of 80% and an alpha of 5%, we calculated the detectable range of OR with sufficient power for the outcome of interest. The significance threshold was set at *p* < 0.05/6 = 0.008 after applying Bonferroni correction for multiple MR tests.

## Results

### Heritability Estimates and Genetic Correlations

Common SNPs (∼1.1 million, EUR phase 3 HapMap) cumulatively explained 1.86% of the total heritability of LBP, suggesting the small effects of SNPs in the genetic contribution to complex disorders. In the GWAS of LBP, genomic inflation factor (λ*_*GC*_* = 1.096) demonstrated slight inflation; with the intercept (1.035) being close to 1, the inflation should be attributed to the polygenic genetic architecture. As shown in Table 1, the heritability estimate on the observed scale, genomic inflation factor, and LDSC intercept for AD, PD, and ALS in this study were similar to those in the original GWASs. Moreover, all these statistics satisfied the following criteria, heritability *H*^2^/SE > 4, mean χ*^2^* > 1.02 and intercepts between 0.9 and 1.1, indicating the suitability and reliability for estimating genetic correlations.

**TABLE 1:** Heritability estimates based on single-nucleotide polymorphisms for low back pain and three neurodegenerative diseases.

**Traits**	***H*^2^(SE)**	***λ_*GC*_***	**Mean χ*^2^***	**Intercept (SE)**
Low back pain	1.86% (0.32%)	1.096	1.102	1.035 (0.008)
Alzheimer’s disease	7.13% (1.14%)	1.093	1.118	1.030 (0.008)
Parkinson’s disease	1.85% (0.18%)	1.090	1.137	0.985 (0.007)
Amyotrophic lateral sclerosis	3.17% (0.70%)	1.044	1.071	1.020 (0.007)

*H^2^, heritability estimate on the observed scale; SE, standard error; λ_*GC*_, genomic inflation factor.*

There was no evidence for the genetic correlation between LBP and AD (*Rg* = −0.033, *p* = 0.766). As detailed in Table 2, correlations between LBP and PD (*Rg* = −0.079, *p* = 0.279) and ALS (*Rg* = 0.069, *p* = 0.583) did not reach nominal significance, either.

**TABLE 2:** Genetic correlations between low back pain and three neurodegenerative diseases.

**Phenotypes**	***R*_*g*_ (95% CI)**	***p*-value**
Alzheimer’s disease	−0.033 (−0.252, 0.186)	0.766
Parkinson’s disease	−0.079 (−0.223, 0.064)	0.279
Amyotrophic lateral sclerosis	0.069 (−0.177, 0.315)	0.583

*R_*g*_, genetic correlation estimate; CI, confidence interval.*

### Bidirectional MR Analyses

**FIGURE 1:**
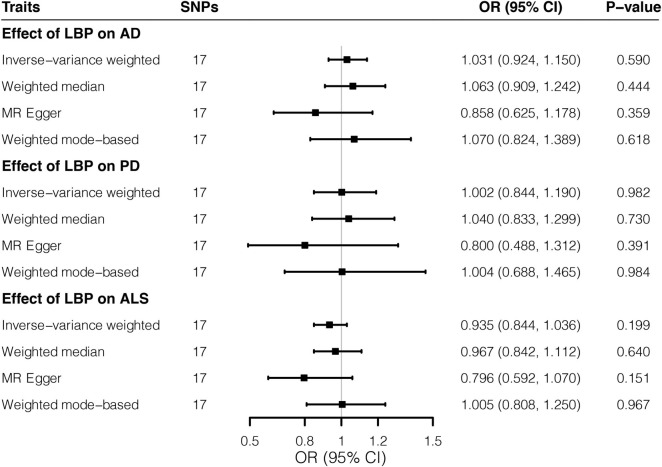
Effects of low back pain on three neurodegenerative diseases by Mendelian randomization analyses. Relative odds of the occurrence of three neurodegenerative diseases given exposure to low back pain were generated by three Mendelian randomization methods and presented in forest plots. AD, Alzheimer’s disease; CI, confidence interval; ALS, amyotrophic lateral sclerosis; LBP, low back pain; OR, odds ratio; PD, Parkinson’s disease; SNP, Single-nucleotide polymorphism.

Overall, MR estimates suggested that genetically predicted higher risks of LBP were not associated with the liability to AD, PD, or ALS. By the IVW approach, genetically predicted predisposition to LBP was not associated with the risk of AD (*OR* = 1.031; 95% CI, 0.924–1.150; *p* = 0.590). Likewise, causal effects of LBP on PD (*OR* = 1.002; 95% CI, 0.844–1.190; *p* = 0.982) and ALS (*OR* = 0.935; 95% CI, 0.844–1.036; *p* = 0.199) did not reach significance threshold in the main analysis. Complementary MR methods provided consistent results (Figure 1 and Supplementary Figure 1). Notably, our analysis might be underpowered (Supplementary Table 4) to detect small causal effects given the small proportion of variance explained by instrumental SNPs. No unbalanced horizontal pleiotropy (all *p* > 0.05) was indicated by MR-Egger regression intercepts (Supplementary Table 5). Cochran’s *Q* tests provided no evidence for the existence of heterogeneity (Supplementary Table 6), whereas leave-one-out plots (Supplementary Figure 2) did not identify any outlier variants.

**FIGURE 2:**
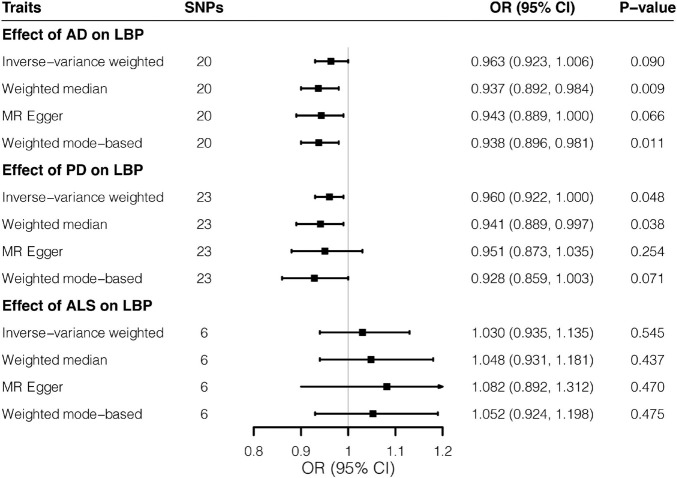
Effects of three neurodegenerative diseases on low back pain by Mendelian randomization analyses. Relative odds of the occurrence of low back pain given exposure to three neurodegenerative diseases were generated by three Mendelian randomization methods and presented in forest plots. AD, Alzheimer’s disease; CI, confidence interval; ALS, amyotrophic lateral sclerosis; LBP, low back pain; OR, odds ratio; PD, Parkinson’s disease; SNP, Single-nucleotide polymorphism.

In the reverse direction, MR analyses did not support the effects of neurodegenerative diseases on LBP. A one-unit increase in log-OR of AD was not associated with change in risks of LBP (*OR* = 0.963; 95% CI, 0.923–1.006; *p* = 0.090) by the IVW method, whereas the weighted median estimate reached nominal significance, albeit failing the Bonferroni-corrected threshold (*p* = 0.009 > 0.05/6). Similarly, as shown in Figure 2, the relationship between PD and LBP (*OR* = 0.960; 95% CI, 0.922–1.000; *p* = 0.048) reached nominal significance. However, there was no evidence for the association of ALS with LBP (*OR* = 1.030; 95% CI, 0.935–1.135; *p* = 0.545). According to scatter plots (Supplementary Figure 3) and leave-one-out plots (Supplementary Figure 4), no evident outliers existed, while additional analyses (Supplementary Tables 5, 6) demonstrated no horizontal pleiotropy or heterogeneity.

## Discussion

In this study, we did not find evidence supporting genetic correlations or causations between non-specific LBP and three common neurodegenerative diseases. Back pain has been commonly studied as a self-reported symptom (Suri et al., 2018; Freidin et al., 2019) and studied in spine-related diseases like lumbar spinal stenosis (Suri et al., 2021). For example, a previous GWAS (Freidin et al., 2019) of self-reported back pain in 509,000 Europeans identified three significant loci (*p* < 5 × 10^–8^), but genetic correlation estimates between back pain and AD (*R*_*g*_ = 0.115, *p* = 0.147), PD (*R*_*g*_ = 0.029, *p* = 0.586), and ALS (*R*_*g*_ = 0.166, *p* = 0.030) all failed Bonferroni-corrected significance. Notably, only a small part of LBP has clear causes and can be classified into specific diseases; however, there exists the majority with unknown mechanisms. Such LBP has been seen as an entity itself in the electronic health record, and as a complex trait, GWAS and related tools are likely to be powerful to disentangle the genetic underpinnings. Here, we employed LDSC and MR to elucidate their relationships based on biobank association data of LBP and the most up-to-date GWASs of AD, PD, and ALS.

Observational studies exploring the relationship between LBP and neurodegenerative diseases have been conducted before (Broetz et al., 2007; Aggarwal et al., 2010; Miller et al., 2013; Udeh-Momoh et al., 2019; Silveira Barezani et al., 2020). Several studies reported a high occurrence of LBP during the non-interventional course of AD (Udeh-Momoh et al., 2019), and the interventional diagnostic and therapeutic procedure of AD (Landen et al., 2013; Alcolea et al., 2014). Similarly, LBP was a common complaint during the treatment of ALS (Aggarwal et al., 2010; Miller et al., 2013). We could not tell whether there are causal mechanisms underlying such findings, given the complexity of insufficiently controlled factors in traditional epidemiology. Regarding the potential role of LBP in PD, in a recent questionnaire-based study (Silveira Barezani et al., 2020), about 40% patients reported the onset of LBP before the diagnosis of PD, and higher pain scores were associated with more advanced stage and rating scales of PD. The interaction of LBP and PD undoubtedly leads to more difficulty and disability in daily activities. Besides, both PD and ALS extensively involved neural and musculoskeletal systems with a variety of manifestations and unbalanced musculoskeletal dynamics due to gait abnormality, posture alteration and chronic joint trauma in the progressive course were likely to result in LBP (Ozturk and Kocer, 2018; Duncan et al., 2019). The vicious cycle of LBP and neurodegenerative diseases should have a severe influence on the life quality of patients. Identifying possible links underlying LBP, AD, PD, and ALS from the perspective of genetic correlations would provide more informative knowledge and ultimately benefit in developing effective interventions. In this study, we found no evidence for the causal effects of LBP on neurodegenerative diseases, neither did the reverse effects reach Bonferroni-corrected threshold (*p* < 0.05/6 = 0.008). The effects of AD and PD on LBP reached nominal significance, and interestingly, the genetic predisposition to AD and PD seemed to be associated with the lower occurrence of LBP in this study. The findings failed to agree with previous observational studies and were against common intuition to a certain extent. Notably, it may as well be common sense that more environmental components (i.e., sedentary behaviors) rather than genetic underpinnings would underlie the liability to LBP when compared with neurodegenerative diseases. In the current statistical model of MR, however, both the exposures and outcomes of interest were genetically predicted “ideal” traits, which were proxied by common variants without taking account of other factors. Undoubtedly, MR estimates alone were not enough. Triangulating evidence across multiple lines of studies is necessary to shed light on relationships between complex traits.

The major strength of this study was the utilization of the state-of-the-art tools, LDSC and MR, to explore the relationships between complex disorders. Using millions of summary-level statistics from hundreds of thousands of participants, LDSC was a powerful tool to estimate the genetic correlation. Based on a subset of instrumental SNPs strongly associated with the exposure-trait of interest, MR was capable of estimating the causal effect on the outcome-trait concerned, while circumventing reverse causation and minimizing biases by confounders. There were also several limitations. Firstly, LBP was defined by electronic health record codes with more reliability and less misclassification, but we could not tell whether the relationship existed between chronic LBP and neurodegenerative diseases. LBP was studied as a whole, without separating the acute and chronic type as generally included in self-reported questionnaires. Neither did we differentiate between subgroups of neurodegenerative diseases like AD subtypes based on neuropathology and neuroimaging, PD subtypes by age at onset (i.e., early-onset and late-onset), and ALS subgroups classified by site of onset (i.e., bulbar and spinal). Secondly, gender differences in the prevalence of LBP and three neurodegenerative diseases have been proposed; however, we could not address the meaningful question since no sex-stratified association data were available. Lastly, this study was based on datasets from European-ancestry GWASs, and great attention should be paid when generalizing the findings to the other populations.

In summary, our results provided no evidence for the genetic correlations between LBP and three common neurodegenerative diseases, AD, PD, and ALS.

## Data Availability Statement

The original contributions presented in the study are included in the article/Supplementary Material, further inquiries can be directed to the corresponding author/s.

## Author Contributions

PW, BW, and KX conceptualized the study. PW, BD, RY, and WZ contributed to acquisition and analysis of data and validation and visualization of results. PW, BD, XL, and YD took part in drafting and reviewing the main manuscript. BW and KX played a role in project administration and funding acquisition. All authors contributed to the article and approved the final version of the manuscript.

## Conflict of Interest

The authors declare that the research was conducted in the absence of any commercial or financial relationships that could be construed as a potential conflict of interest.

## Publisher’s Note

All claims expressed in this article are solely those of the authors and do not necessarily represent those of their affiliated organizations, or those of the publisher, the editors and the reviewers. Any product that may be evaluated in this article, or claim that may be made by its manufacturer, is not guaranteed or endorsed by the publisher.

## Footnotes

1
https://data.broadinstitute.org/alkesgroup/LDSCORE/
