# Supplementary material for: Joint Analysis of Genome-Wide Association Data Reveals No Genetic Correlations Between Low Back Pain and Neurodegenerative Diseases
Source: Front Genet. 2021 Sep 22;12:744299. doi: 10.3389/fgene.2021.744299 (PMC8493037; doi:10.3389/fgene.2021.744299)
Supplement: Supplementary file 1 [file Data_Sheet_1.pdf]

Supplementary Material

Supplementary Table 1. Brief description of source datasets utilized in this study

Supplementary Table 2. Summary statistics utilized in the Mendelian randomization analysis of low back pain on neurodegenerative diseases

Supplementary Table 3. Summary statistics utilized in the Mendelian randomization analysis of neurodegenerative diseases on low back pain

Supplementary Table 4. Mendelian randomization analysis power estimation

Supplementary Table 5. MR-Egger regression intercepts examining unbalanced horizontal pleiotropy

Supplementary Table 6. Heterogeneity assessment by Cochran's Q statistics

Supplementary Figure 1. Scatter plots in the Mendelian randomization analyses of low back pain on neurodegenerative diseases

Supplementary Figure 2. Leave-one-out plots in the Mendelian randomization analyses of low back pain on neurodegenerative diseases

Supplementary Figure 3. Scatter plots in the Mendelian randomization analyses of neurodegenerative diseases on low back pain

Supplementary Figure 4. Leave-one-out plots in the Mendelian randomization analyses of neurodegenerative diseases on low back pain

Supplementary Table 1. Brief description of source datasets utilized in this study

Traits	Sample size	Cases	Controls	Ancestry	Access Link
Low back pain	177,860	13,178	164,682	European	https://r5.finngen.fi/
Alzheimer's disease	63,926	21,982	41,944	European	https://www.niagads.org/datasets/ng00075
Parkinson's disease	482,730	33,674	449,056	European	https://pdgenetics.org/resources
Amyotrophic lateral sclerosis	80,610	20,806	59,804	European	http://als.umassmed.edu/

Supplementary Table 2. Summary statistics utilized in the Mendelian randomization analysis of low back pain on neurodegenerative diseases

Trait	SNP	Coordinate (GRCh37)	EA	OA	EAF	R^2	F	Association with LBP			Association with outcome trait		
								Beta	SE	*P*-value	Beta	SE	*P*-value
AD	rs6426282	1:246310585	C	T	0.196	0.002	22.3	-0.080	0.017	2.27×10^{-6}	0.006	0.021	0.783
AD	rs56091004	6:3744847	T	C	0.061	0.002	23.9	0.140	0.029	1.05×10^{-6}	-0.039	0.028	0.171
AD	rs9469835	6:34611001	G	A	0.330	0.002	25.3	0.073	0.015	4.83×10^{-7}	-0.005	0.015	0.759
AD	rs112412403	6:129060572	A	G	0.038	0.002	21.7	0.169	0.036	3.15×10^{-6}	-0.041	0.070	0.555
AD	rs147356029	7:4042724	A	G	0.018	0.002	21.5	0.238	0.051	3.46×10^{-6}	-0.040	0.045	0.376
AD	rs143783757	7:79133344	G	A	0.021	0.002	21.2	-0.220	0.048	4.06×10^{-6}	-0.023	0.068	0.732
AD	rs1481789	8:72040468	A	G	0.159	0.002	24.1	0.091	0.019	9.94×10^{-7}	0.008	0.017	0.661
AD	rs4284332	10:73733952	C	T	0.557	0.002	22.6	0.065	0.014	2.10×10^{-6}	0.006	0.015	0.690
AD	rs231908	11:2751660	G	A	0.755	0.003	27.8	-0.083	0.016	1.42×10^{-7}	-0.024	0.018	0.179
AD	rs60127327	12:54627857	G	A	0.481	0.002	22.4	-0.064	0.014	2.42×10^{-6}	-0.031	0.015	0.034
AD	rs941157	12:90776124	T	C	0.412	0.002	22.1	-0.064	0.014	2.76×10^{-6}	-0.006	0.014	0.692

AD	rs225880	14:30481804	C	T	0.786	0.002	22.7	0.078	0.016	1.94×10^{-6}	-0.017	0.019	0.375
AD	rs2022845	14:66343168	T	G	0.309	0.002	22.5	-0.069	0.015	2.21×10^{-6}	-0.011	0.015	0.448
AD	rs72726942	15:31876216	T	G	0.026	0.002	21.7	0.202	0.043	3.17×10^{-6}	0.008	0.041	0.841
AD	rs56807215	16:87951030	A	T	0.099	0.002	21.2	-0.106	0.023	4.22×10^{-6}	-0.022	0.033	0.504
AD	rs62097992	18:50835863	C	T	0.412	0.002	23.5	0.067	0.014	1.17×10^{-6}	-0.015	0.014	0.298
AD	rs113679764	19:17486898	A	G	0.096	0.002	21.9	0.108	0.023	2.99×10^{-6}	0.042	0.032	0.195
PD	rs6426282	1:246310585	C	T	0.196	0.002	22.3	-0.080	0.017	2.27×10^{-6}	0.035	0.032	0.274
PD	rs56091004	6:3744847	T	C	0.061	0.002	23.9	0.140	0.029	1.05×10^{-6}	0.040	0.038	0.289
PD	rs9469835	6:34611001	G	A	0.330	0.002	25.3	0.073	0.015	4.83×10^{-7}	-0.012	0.018	0.504
PD	rs112412403	6:129060572	A	G	0.038	0.002	21.7	0.169	0.036	3.15×10^{-6}	-0.006	0.075	0.932
PD	rs147356029	7:4042724	A	G	0.018	0.002	21.5	0.238	0.051	3.46×10^{-6}	0.015	0.068	0.825
PD	rs143783757	7:79133344	G	A	0.021	0.002	21.2	-0.220	0.048	4.06×10^{-6}	0.022	0.085	0.797
PD	rs1481789	8:72040468	A	G	0.159	0.002	24.1	0.091	0.019	9.94×10^{-7}	0.030	0.029	0.301
PD	rs4284332	10:73733952	C	T	0.557	0.002	22.6	0.065	0.014	2.10×10^{-6}	-0.015	0.023	0.506
PD	rs231908	11:2751660	G	A	0.755	0.003	27.8	-0.083	0.016	1.42×10^{-7}	-0.026	0.028	0.345
PD	rs60127327	12:54627857	G	A	0.481	0.002	22.4	-0.064	0.014	2.42×10^{-6}	-0.005	0.020	0.808
PD	rs941157	12:90776124	T	C	0.412	0.002	22.1	-0.064	0.014	2.76×10^{-6}	-0.042	0.022	0.062

PD	rs225880	14:30481804	C	T	0.786	0.002	22.7	0.078	0.016	1.94×10^{-6}	-0.019	0.029	0.520
PD	rs2022845	14:66343168	T	G	0.309	0.002	22.5	-0.069	0.015	2.21×10^{-6}	-0.001	0.023	0.981
PD	rs72726942	15:31876216	T	G	0.026	0.002	21.7	0.202	0.043	3.17×10^{-6}	-0.097	0.062	0.118
PD	rs56807215	16:87951030	A	T	0.099	0.002	21.2	-0.106	0.023	4.22×10^{-6}	0.089	0.037	0.017
PD	rs62097992	18:50835863	C	T	0.412	0.002	23.5	0.067	0.014	1.17×10^{-6}	0.028	0.020	0.155
PD	rs113679764	19:17486898	A	G	0.096	0.002	21.9	0.108	0.023	2.99×10^{-6}	0.005	0.042	0.914
ALS	rs6426282	1:246310585	C	T	0.196	0.002	22.3	-0.080	0.017	2.27×10^{-6}	0.024	0.019	0.209
ALS	rs56091004	6:3744847	T	C	0.061	0.002	23.9	0.140	0.029	1.05×10^{-6}	0.003	0.024	0.894
ALS	rs9469835	6:34611001	G	A	0.330	0.002	25.3	0.073	0.015	4.83×10^{-7}	-0.006	0.014	0.663
ALS	rs112412403	6:129060572	A	G	0.038	0.002	21.7	0.169	0.036	3.15×10^{-6}	-0.051	0.053	0.344
ALS	rs147356029	7:4042724	A	G	0.018	0.002	21.5	0.238	0.051	3.46×10^{-6}	-0.089	0.045	0.045
ALS	rs143783757	7:79133344	G	A	0.021	0.002	21.2	-0.220	0.048	4.06×10^{-6}	0.008	0.064	0.904
ALS	rs1481789	8:72040468	A	G	0.159	0.002	24.1	0.091	0.019	9.94×10^{-7}	-0.007	0.016	0.676
ALS	rs4284332	10:73733952	C	T	0.557	0.002	22.6	0.065	0.014	2.10×10^{-6}	-0.006	0.014	0.667
ALS	rs231908	11:2751660	G	A	0.755	0.003	27.8	-0.083	0.016	1.42×10^{-7}	-0.006	0.017	0.707
ALS	rs60127327	12:54627857	G	A	0.481	0.002	22.4	-0.064	0.014	2.42×10^{-6}	0.001	0.014	0.956
ALS	rs941157	12:90776124	T	C	0.412	0.002	22.1	-0.064	0.014	2.76×10^{-6}	-0.005	0.014	0.746

ALS	rs225880	14:30481804	C	T	0.786	0.002	22.7	0.078	0.016	1.94×10^{-6}	-0.021	0.018	0.242
ALS	rs2022845	14:66343168	T	G	0.309	0.002	22.5	-0.069	0.015	2.21×10^{-6}	-0.016	0.014	0.257
ALS	rs72726942	15:31876216	T	G	0.026	0.002	21.7	0.202	0.043	3.17×10^{-6}	0.013	0.038	0.743
ALS	rs56807215	16:87951030	A	T	0.099	0.002	21.2	-0.106	0.023	4.22×10^{-6}	0.043	0.028	0.122
ALS	rs62097992	18:50835863	C	T	0.412	0.002	23.5	0.067	0.014	1.17×10^{-6}	0.006	0.014	0.655
ALS	rs113679764	19:17486898	A	G	0.096	0.002	21.9	0.108	0.023	2.99×10^{-6}	-0.008	0.032	0.816

Note: As for instrumental SNPs for the exposure, proportion of variance explained was calculated using formula**:** $R^2 = 2 \times \text{EAF} \times (1\text{-EAF}) \times \text{Beta}^2$. Strength of individual SNP was approximated by the formula: F-statistic = $(\text{Beta/SE})^2$, where $F < 10$ was seen as a weak instrument.

Abbreviations**:** AD, Alzheimer's disease; ALS, amyotrophic lateral sclerosis; EA, effect allele; EAF, effect allele frequency of instrumental SNP associated with the exposure; LBP, low back pain; SE, standard error; OA, other allele; PD, Parkinson's disease; SNP, single nucleotide polymorphism.

Supplementary Table 3. Summary statistics utilized in the Mendelian randomization analysis of neurodegenerative diseases on low back pain

Trait	SNP	Coordinate (GRCh37)	EA	OA	EAF	R^2	F	Association with exposure trait			Association with LBP		
								Beta	SE	*P*-value	Beta	SE	*P*-value
AD	rs679515	1:207750568	C	T	0.828	0.006	70.4	-0.151	0.018	1.55×10^{-16}	-0.005	0.017	0.777
AD	rs6733839	2:127892810	T	C	0.380	0.014	126.9	0.169	0.015	4.02×10^{-28}	-0.006	0.014	0.700
AD	rs114812713	6:41034000	C	G	0.028	0.005	48.0	0.298	0.043	4.47×10^{-12}	0.188	0.068	0.006
AD	rs9381563	6:47432637	T	C	0.322	0.003	29.9	-0.082	0.015	2.93×10^{-8}	-0.013	0.016	0.420
AD	rs11767557	7:143109139	C	T	0.218	0.004	32.7	-0.103	0.018	1.56×10^{-8}	-0.025	0.018	0.176
AD	rs73223431	8:27219987	T	C	0.346	0.004	39.3	0.094	0.015	8.34×10^{-10}	-0.021	0.014	0.142
AD	rs867230	8:27468503	A	C	0.598	0.009	69.1	0.133	0.016	3.49×10^{-17}	-0.013	0.014	0.340
AD	rs11257242	10:11721119	G	C	0.651	0.003	31.4	0.084	0.015	4.64×10^{-8}	-0.010	0.014	0.459
AD	rs3740688	11:47380340	T	G	0.543	0.004	45.1	0.094	0.014	9.70×10^{-11}	0.006	0.015	0.704
AD	rs1582763	11:60021948	A	G	0.363	0.007	67.2	-0.123	0.015	1.19×10^{-16}	0.009	0.015	0.548
AD	rs3851179	11:85868640	C	T	0.629	0.007	64.0	0.120	0.015	5.81×10^{-16}	0.018	0.014	0.197
AD	rs12590654	14:92938855	A	G	0.337	0.004	32.3	-0.091	0.016	8.73×10^{-9}	0.008	0.015	0.601

AD	rs12151021	19:1050874	G	A	0.662	0.005	39.6	-0.107	0.017	2.56×10^{-10}	-0.012	0.015	0.431
AD	rs111278137	19:45215081	A	G	0.014	0.006	44.6	-0.474	0.071	3.20×10^{-11}	0.070	0.068	0.305
AD	rs147711004	19:45337918	A	G	0.029	0.072	941.0	1.135	0.037	1.00×10^{-200}	-0.071	0.034	0.036
AD	rs7412	19:45412079	T	C	0.063	0.026	226.9	-0.467	0.031	6.40×10^{-53}	0.037	0.030	0.219
AD	rs1081105	19:45412955	C	A	0.031	0.053	458.3	0.942	0.044	1.51×10^{-103}	-0.071	0.048	0.142
AD	rs72654445	19:45417200	A	G	0.011	0.006	44.9	-0.543	0.081	2.27×10^{-11}	-0.073	0.066	0.273
AD	rs139136389	19:45427136	T	C	0.026	0.012	33.8	-0.494	0.085	6.43×10^{-9}	0.121	0.110	0.272
AD	rs150685845	19:45675180	G	A	0.013	0.008	73.2	0.556	0.065	6.62×10^{-18}	0.043	0.050	0.388
PD	rs35749011*	1:155135036	A	G	0.019	0.021	129.5	0.751	0.066	5.02×10^{-30}	-0.052	0.033	0.111
PD	rs823106	1:205656453	C	G	0.849	0.006	38.5	-0.149	0.024	4.10×10^{-10}	0.011	0.019	0.557
PD	rs6741007	2:135537119	G	T	0.451	0.008	46.7	-0.123	0.018	2.09×10^{-12}	0.006	0.014	0.657
PD	rs4613239	2:169119609	G	C	0.133	0.007	50.7	0.178	0.025	6.21×10^{-13}	0.029	0.022	0.194
PD	rs4488803	3:58218352	A	G	0.375	0.006	32.5	-0.114	0.020	1.08×10^{-8}	0.002	0.014	0.882
PD	rs10513789	3:182760073	G	T	0.183	0.008	52.9	-0.160	0.022	3.18×10^{-13}	-0.019	0.017	0.272
PD	rs34311866	4:951947	C	T	0.196	0.016	97.4	0.227	0.023	7.97×10^{-23}	-0.020	0.017	0.225
PD	rs4698412	4:15737348	A	G	0.553	0.008	54.9	0.126	0.017	7.05×10^{-14}	-0.018	0.014	0.193
PD	rs7695720	4:77183300	C	A	0.209	0.005	36.0	-0.126	0.021	1.53×10^{-9}	0.015	0.016	0.365

PD	rs356203	4:90666041	T	C	0.617	0.027	177.8	-0.240	0.018	3.01×10^{-41}	-0.006	0.014	0.696
PD	rs75646569	5:60345424	G	T	0.112	0.007	50.6	0.192	0.027	5.62×10^{-13}	0.024	0.023	0.286
PD	rs35265698	6:32561334	G	C	0.155	0.010	44.4	-0.200	0.030	3.93×10^{-11}	0.010	0.019	0.581
PD	rs858295	7:23245569	G	A	0.395	0.005	33.4	-0.104	0.018	3.83×10^{-9}	0.015	0.014	0.291
PD	rs620490	8:16697579	G	T	0.276	0.006	37.9	-0.117	0.019	6.46×10^{-10}	-0.004	0.014	0.762
PD	rs144814361	10:121410917	T	C	0.017	0.007	42.1	0.441	0.068	9.07×10^{-11}	-0.013	0.088	0.884
PD	rs329647	11:133764666	C	G	0.666	0.006	39.4	-0.113	0.018	1.94×10^{-10}	0.013	0.018	0.461
PD	rs75505347	12:40885549	T	C	0.020	0.006	34.2	0.392	0.067	6.12×10^{-9}	-0.061	0.057	0.289
PD	rs10847864	12:123326598	T	G	0.363	0.008	49.8	0.127	0.018	9.81×10^{-13}	0.006	0.014	0.698
PD	rs4774417	15:61993702	A	G	0.740	0.004	30.5	0.105	0.019	4.63×10^{-8}	-0.019	0.015	0.202
PD	rs12934900	16:30923602	T	A	0.657	0.007	45.9	0.122	0.018	4.33×10^{-11}	0.007	0.014	0.619
PD	rs10451230	17:16035225	T	A	0.565	0.005	28.4	-0.096	0.018	4.42×10^{-8}	0.013	0.014	0.347
PD	rs58879558*	17:44095467	C	T	0.223	0.020	90.6	-0.238	0.025	1.36×10^{-21}	0.035	0.021	0.096
PD	rs4588066	18:40672964	A	G	0.326	0.005	34.0	0.105	0.018	4.45×10^{-9}	0.001	0.014	0.962
ALS	rs10463311	5:150410835	T	C	0.744	0.003	28.2	-0.085	0.016	4.00×10^{-8}	0.011	0.015	0.492
ALS	rs3849943	9:27543382	T	C	0.752	0.012	121.0	-0.176	0.016	3.77×10^{-30}	-0.003	0.018	0.885
ALS	rs142321490	12:58676132	C	G	0.018	0.004	38.6	0.317	0.051	6.15×10^{-10}	0.016	0.042	0.704

ALS	rs74654358	12:64881967	A	G	0.047	0.004	33.9	0.198	0.034	4.66×10^{-9}	0.008	0.035	0.826
ALS	rs12973192	19:17753239	G	C	0.325	0.006	65.1	0.121	0.015	3.92×10^{-15}	0.008	0.014	0.588
ALS	rs75087725	21:45753117	A	C	0.015	0.008	59.1	0.515	0.067	1.85×10^{-14}	0.025	0.047	0.590

Note: As for instrumental SNPs for the exposure, proportion of variance explained was calculated using the formula: $R^2 = 2 \times \text{EAF} \times (1\text{-EAF}) \times \text{Beta}^2$. Strength of individual SNP was approximated by the formula: F-statistic = $(\text{Beta/SE})^2$, where $F < 10$ was seen as a weak instrument. Effect allele frequency for instrumental variable of AD was not available in the original GWAS, and hence was retrieved by searching LDlink in the EUR sub-population (1000 Genomes Phase 3).

* In the summary statistics of low back pain two proxy SNPs were utilized for rs35749011 (rs35682329, chr1:155121143, $r^2 = 1$) and rs58879558 (rs62062271, chr17: 44091988, $r^2 = 0.99$).

Abbreviations**:** AD, Alzheimer's disease; ALS, amyotrophic lateral sclerosis; EA, effect allele; EAF, effect allele frequency of instrumental SNP associated with the exposure; LBP, low back pain; SE, standard error; OA, other allele; PD, Parkinson's disease; SNP, single nucleotide polymorphism.

Supplementary Table 4. Mendelian randomization analysis power estimation

Exposure	No. of SNPs	PVE	Outcome	Outcome sample size	*K*	OR detected with adequate power (>80%)
LBP	17	0.036	AD	63,926	0.344	≤0.882, or ≥ 1.129
LBP	17	0.036	PD	482,730	0.070	≤0.917, or ≥ 1.084
LBP	17	0.036	ALS	80,610	0.258	≤0.885, or ≥ 1.122
AD	20	0.262	LBP	177,860	0.074	≤0.951, or ≥ 1.050
PD	23	0.206	LBP	177,860	0.074	≤0.944, or ≥ 1.056
ALS	6	0.036	LBP	177,860	0.074	≤0.867, or ≥ 1.136

Note: Assuming a power of 80% and an alpha of 5%, we calculated the minimum detectable OR using the web application, mRnd (https://shiny.cnsgenomics.com/mRnd/) with input parameters: (1) PVE, proportion of variance explained for the association between instrumental SNPs with the exposure; (2) the outcome sample size; and (3) K, proportion of cases in the association study of the outcome of interest.

Abbreviations: AD, Alzheimer's disease; ALS, amyotrophic lateral sclerosis; LBP, low back pain; PD, Parkinson's disease; SNP, Single-nucleotide polymorphism.

Supplementary Table 5. MR-Egger regression intercepts examining unbalanced horizontal pleiotropy

Exposures and outcomes	Number of SNPs	MR-Egger regression		
		Intercept	Standard error	*P*-value
LBP on AD	17	0.017	0.014	0.246
LBP on PD	17	0.021	0.022	0.357
LBP on ALS	17	0.015	0.013	0.273
AD on LBP	20	0.007	0.007	0.307
PD on LBP	23	0.002	0.007	0.792
ALS on LBP	6	-0.010	0.017	0.598

Abbreviations: AD, Alzheimer’s disease; ALS, amyotrophic lateral sclerosis; LBP, low back pain; PD, Parkinson’s disease; SNP, Single-nucleotide polymorphism.

Supplementary Table 6. Heterogeneity assessment by Cochran's Q statistics

Exposures and outcomes	Number of SNPs	Cochran's *Q* test		
		Q-statistic	I^2	*P*-value
LBP on AD	17	14.5	0	0.564
LBP on PD	17	19.4	17.6%	0.248
LBP on ALS	17	11.1	0	0.805
AD on LBP	20	25.5	25.5%	0.144
PD on LBP	23	16.3	0	0.801
ALS on LBP	6	0.9	0	0.970

Note: I^2 statistic measured the percentage of variation across instrumental variants that is due to heterogeneity, $I^2 = (Q-df)/Q \times 100\%$, where df = N-1, df was degree of freedom, N was number of instrumental SNPs.

Abbreviations: AD, Alzheimer's disease; ALS, amyotrophic lateral sclerosis; LBP, low back pain; PD, Parkinson's disease; SNP, Single-nucleotide polymorphism.

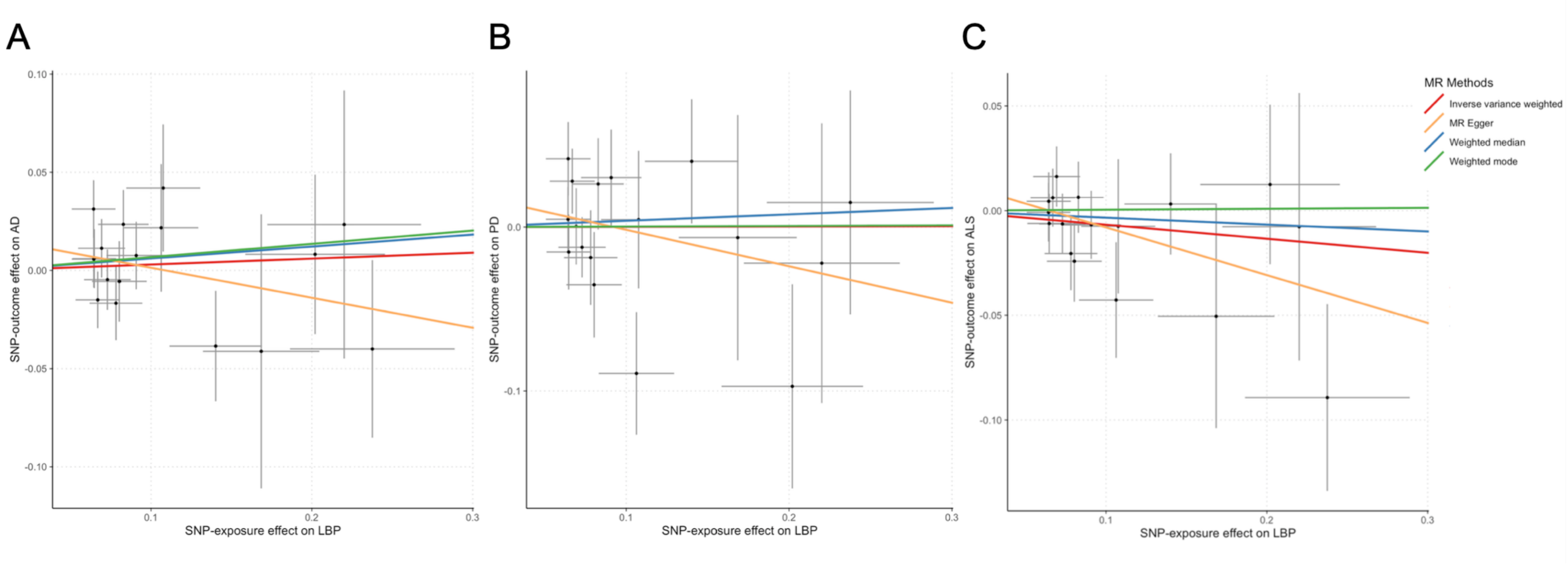

Supplementary Figure 1. Scatter plots in the Mendelian randomization analyses of low back pain on neurodegenerative diseases

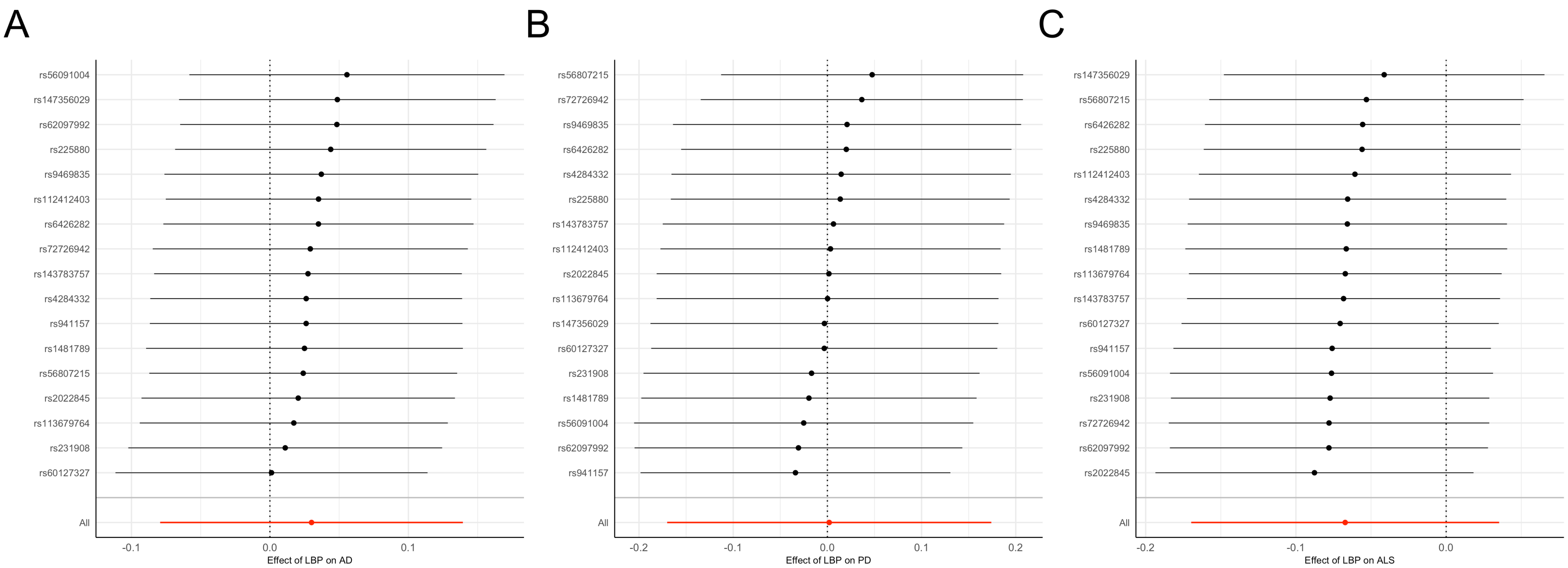

Supplementary Figure 2. Leave-one-out plots in the Mendelian randomization analyses of low back pain on neurodegenerative diseases

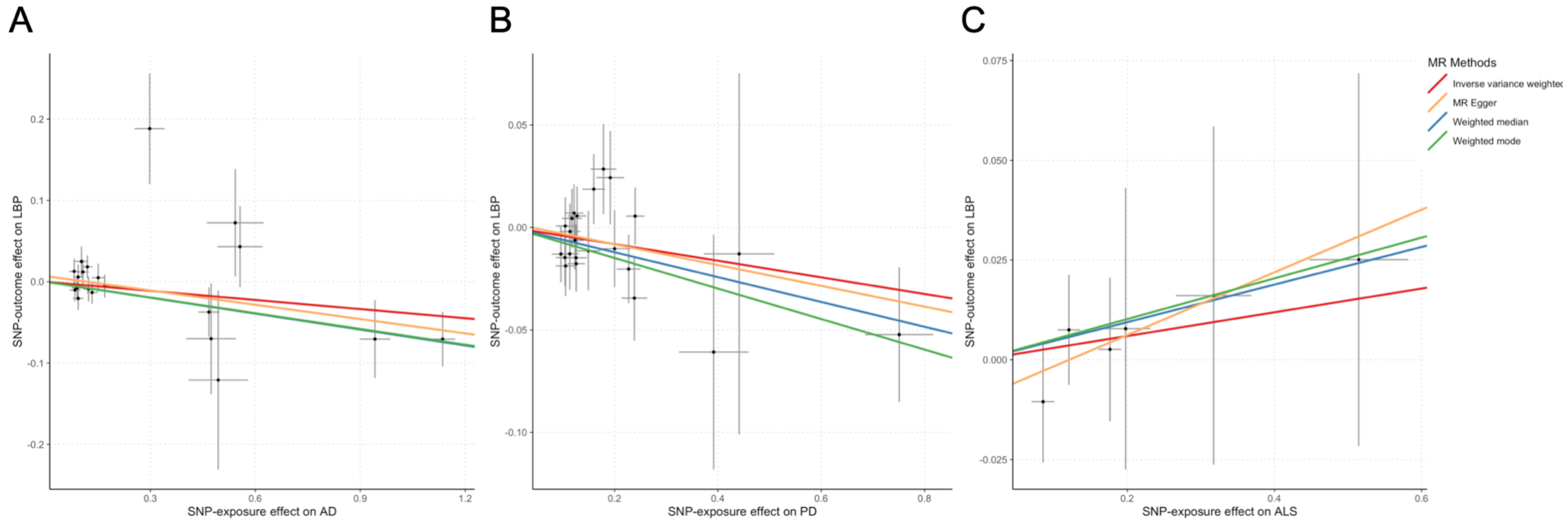

Supplementary Figure 3. Scatter plots in the Mendelian randomization analyses of neurodegenerative diseases on low back pain

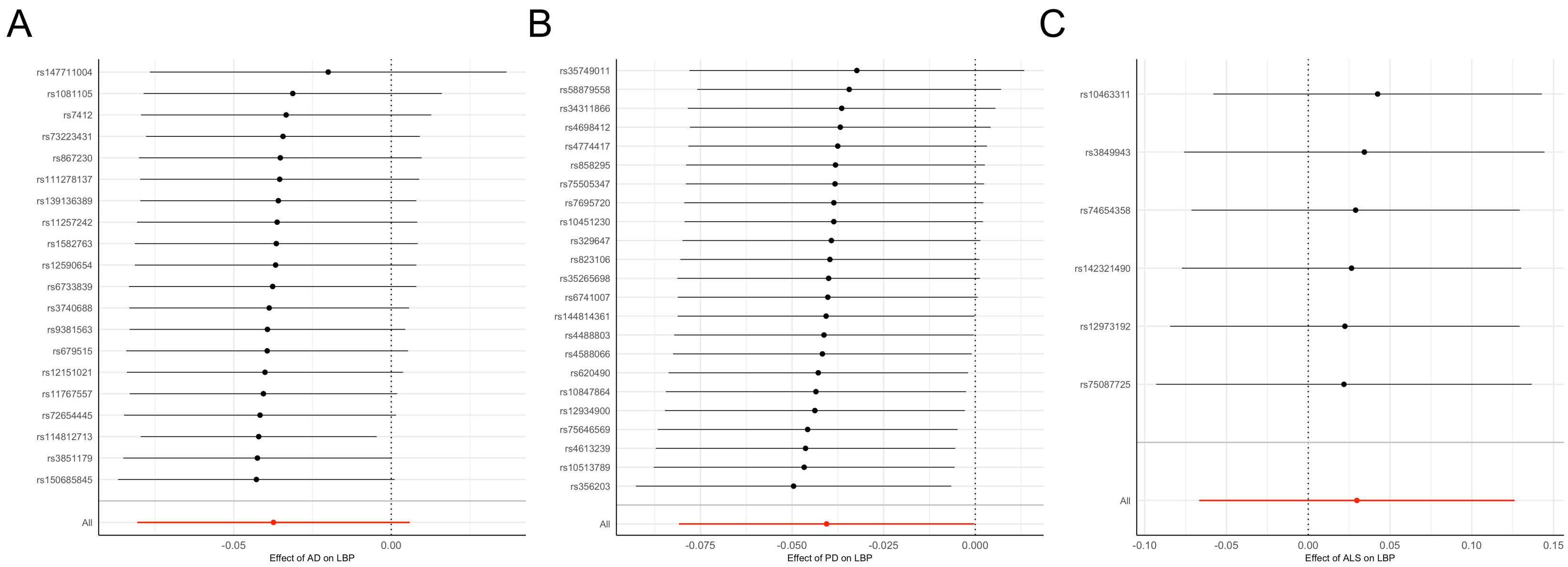

Supplementary Figure 4. Leave-one-out plots in the Mendelian randomization analyses of neurodegenerative diseases on low back pain
